# Supplementary material for: A Likelihood Approach for Real-Time Calibration of Stochastic Compartmental Epidemic Models
Source: PLoS Comput Biol. 2017 Jan 17;13(1):e1005257. doi: 10.1371/journal.pcbi.1005257 (PMC5240920; doi:10.1371/journal.pcbi.1005257)
Supplement: S1 File — (TAR.GZ) [file pcbi.1005257.s014.tar.gz › HSPH_Online-SI-Revision/MSS10/n1-severe/n1-severe_NEW_table-8.pdf]

1	R0	{1.51081, 13.7078, 1.}	{1.24916, 4.05433, 0.43}	{{1.06738, 2.19254, 0.}, {1.47488, 4.21286, 2.}}	{20.7291, 73.1953, 95.}	inf	20.	14.1866	{2.9403, 26.2792}	52.1166	0.35506	newly	8.	30.19	{5., 65.}	291.375	0.525825	Reff	1.3839	1.15007	{0.978733, 1.34771}	19.894	ar	656.	279.46	{70., 553.}	59.7104
2	R0	{1.73182, 11.3873, 3.}	{1.82945, 11.1821, 1.03}	{{1.22544, 3.26445, 0.}, {2.48947, 19.255, 2.}}	{24.917, 47.1082, 67.}	inf	10.	24.4128	{5.42646, 46.4546}	160.354	0.392614	newly	5.	23.09	{7., 42.}	365.8	0.577614	Reff	1.66774	1.72909	{1.18564, 2.30623}	23.3292	ar	653.	631.3	{92., 892.}	34.2175
3	R0	{1.50449, 10.3474, 0.1}	{1.20862, 3.24043, 0.14}	{{1.06738, 2.19254, 0.}, {1.28596, 4.00571, 1.}}	{19.7061, 68.6837, 216.}	inf	24.	6.09011	{1.81947, 10.7988}	74.6245	0.688678	newly	17.	17.48	{2., 35.}	65.5294	0.352384	Reff	1.41873	1.16135	{1.02245, 1.24218}	18.228	ar	651.	196.46	{36., 421.}	69.8771
4	R0	{1.63493, 9.1955, 0.1}	{2.25131, 13.4317, 0.34}	{{1.71608, 9.35432, 0.}, {2.88523, 18.2127, 1.}}	{39.5531, 50.8745, 384.}	inf	6.	8.71003	{2.51344, 15.9064}	76.395	0.260981	newly	4.	7.24	{1., 15.}	121.5	0.388587	Reff	1.61368	2.21504	{1.69292, 2.8188}	39.1335	ar	663.	781.57	{559., 929.}	31.6456
5	R0	{1.53822, 8.05553, 0.1}	{1.97512, 14.285, 0.49}	{{1.38427, 9.44073, 0.}, {2.6365, 18.4042, 1.}}	{32.3105, 78.8212, 516.}	inf	7.	8.47522	{2.62379, 14.8838}	49.6941	0.203318	newly	6.	5.43	{1., 10.}	47.5	0.301868	Reff	1.51515	1.94146	{1.36358, 2.58705}	32.0176	ar	621.	691.15	{50., 901.}	34.9356
6	R0	{1.69374, 17.0793, 0.1}	{1.56312, 15.6801, 0.64}	{{1.21457, 11.4771, 0.}, {1.8951, 19.6444, 2.}}	{13.9163, 17.2719, 636.}	inf	15.	9.56998	{2.79349, 18.1268}	48.5823	0.340656	newly	6.	4.92	{1., 9.}	48.6667	0.342555	Reff	1.64801	1.52851	{1.19402, 1.83934}	13.4528	ar	662.	543.65	{207., 772.}	26.7115
7	R0	{1.42179, 11.8031, 2.}	{1.87588, 14.2498, 0.6}	{{1.40191, 8.35044, 0.}, {2.24732, 19.4475, 1.}}	{32.9368, 33.9836, 71.}	inf	25.	23.7152	{11.4552, 43.181}	41.1161	0.202762	newly	16.	14.57	{7., 25.}	40.6875	0.216867	Reff	1.34786	1.77771	{1.34351, 2.12301}	32.6498	ar	517.	716.32	{483., 868.}	44.7582
8	R0	{1.46683, 6.95875, 0.1}	{1.98795, 11.9767, 0.81}	{{1.17082, 5.71232, 0.}, {2.86501, 18.788, 2.}}	{42.3587, 79.8742, 794.}	inf	9.	11.1221	{2.90736, 20.9542}	65.0242	0.269247	newly	10.	9.16	{2., 19.}	58.2	0.314768	Reff	1.44043	1.94528	{1.15284, 2.79231}	41.7382	ar	653.	673.96	{57., 922.}	35.2251
9	R0	{1.34525, 11.505, 1.}	{2.12229, 15.3375, 0.14}	{{1.78225, 10.5184, 0.}, {2.66225, 19.9254, 1.}}	{57.7618, 36.5344, 88.}	inf	14.	33.3346	{19.8117, 50.5287}	138.74	0.352701	newly	10.	18.9	{10., 27.}	93.6	0.269887	Reff	1.2874	1.98775	{1.67366, 2.46637}	54.4001	ar	532.	805.5	{697., 913.}	51.4098
10	R0	{1.77958, 14.372, 0.1}	{2.3901, 17.0531, 0.}	{{2.01705, 13.609, 0.}, {2.66225, 19.9254, 0.}}	{34.3072, 21.8681, 100.}	inf	20.	48.6433	{29.5727, 69.3319}	143.391	0.36422	newly	5.	25.61	{16., 36.}	412.2	0.686608	Reff	1.68348	2.19058	{1.88754, 2.45355}	30.1217	ar	671.	870.27	{809., 924.}	29.6975
11	R0	{1.60334, 19.9548, 1.}	{1.79666, 14.1742, 0.14}	{{1.39882, 8.26611, 0.}, {2.00049, 16.3049, 0.}}	{17.5965, 28.9687, 94.}	inf	26.	34.2884	{14.9696, 53.0563}	51.719	0.196037	newly	11.	18.85	{11., 28.}	76.4545	0.234055	Reff	1.48149	1.64215	{1.29858, 1.8272}	15.977	ar	617.	678.35	{432., 824.}	25.9335
12	R0	{1.68623, 7.58585, 0.1}	{1.98631, 13.3323, 0.5}	{{1.16434, 8.12562, 0.}, {2.81891, 19.2013, 2.}}	{30.962, 80.6387, 524.}	inf	4.	4.4742	{1.47547, 8.71525}	54.8447	0.228944	newly	7.	3.92	{1., 8.}	68.2857	0.574855	Reff	1.67443	1.97061	{1.15797, 2.80346}	30.794	ar	640.	643.31	{5., 924.}	42.3016
13	R0	{1.82689, 18.0022, 1.}	{1.93355, 14.9412, 0.27}	{{1.39882, 8.35044, 0.}, {2.24732, 19.4475, 1.}}	{15.3391, 19.6771, 79.}	inf	23.	19.368	{8.49859, 30.5359}	34.1752	0.191198	newly	6.	10.75	{3., 18.}	96.1667	0.290598	Reff	1.74285	1.85	{1.35405, 2.145}	15.1284	ar	673.	738.1	{536., 860.}	19.893
14	R0	{1.75911, 9.16883, 0.1}	{1.70406, 15.297, 0.37}	{{1.07469, 9.75208, 0.}, {2.59755, 18.9003, 1.}}	{28.6116, 68.5541, 404.}	inf	4.	3.4729	{1.2123, 7.17115}	51.5899	0.266753	newly	3.	2.62	{0., 5.}	82.6667	0.578296	Reff	1.75031	1.69559	{1.07101, 2.59087}	28.4853	ar	661.	455.15	{3., 902.}	53.9924
15	R0	{1.46168, 11.3859, 2.}	{1.50752, 8.55207, 1.16}	{{1.21937, 3.07157, 0.}, {1.93288, 15.3647, 2.}}	{17.168, 46.1387, 50.}	inf	25.	27.4839	{7.24709, 52.7536}	56.1292	0.272979	newly	14.	27.7	{11., 53.}	109.143	0.293152	Reff	1.32428	1.35733	{1.10616, 1.76488}	15.7016	ar	556.	504.36	{185., 792.}	33.3633
16	R0	{1.53577, 11.7581, 1.}	{1.97148, 10.496, 0.58}	{{1.22544, 5.71232, 0.}, {2.64721, 15.5255, 1.}}	{37.1672, 27.942, 70.}	inf	5.	7.46194	{2.09307, 13.9412}	78.4629	0.26655	newly	2.	8.58	{1., 16.}	345.	0.583275	Reff	1.52041	1.94569	{1.21139, 2.60676}	36.7888	ar	625.	656.97	{18., 917.}	41.3392
17	R0	{1.70698, 9.72924, 0.1}	{2.11207, 10.601, 0.79}	{{1.24751, 5.15387, 0.}, {2.86001, 15.5928, 2.}}	{34.632, 35.4918, 780.}	inf	8.	9.82675	{2.10634, 19.5772}	64.3476	0.264809	newly	5.	11.92	{2., 26.}	165.6	0.372027	Reff	1.68138	2.07454	{1.22543, 2.77695}	34.2249	ar	675.	720.18	{57., 928.}	34.5896
18	R0	{1.5973, 14.931, 0.1}	{1.81958, 14.8581, 0.36}	{{1.12882, 9.35432, 0.}, {2.53075, 19.2972, 1.}}	{29.9017, 22.0678, 400.}	inf	9.	3.37802	{1.37669, 5.92878}	63.1639	0.498782	newly	7.	2.33	{0., 5.}	73.5714	0.800266	Reff	1.57973	1.80929	{1.12251, 2.50477}	30.1203	ar	632.	536.06	{5., 910.}	49.2025
19	R0	{1.87468, 17.004, 1.}	{1.4534, 10.3978, 2.08}	{{1.06738, 2.19254, 0.}, {1.68078, 15.6632, 3.}}	{22.4723, 39.8739, 172.}	inf	36.	23.1017	{5.65625, 43.0024}	44.7209	0.323181	newly	15.	20.99	{7., 40.}	67.5333	0.226841	Reff	1.69659	1.33218	{0.998418, 1.51869}	21.4786	ar	692.	487.81	{129., 711.}	30.909
20	R0	{1.84715, 12.531, 0.1}	{2.01436, 16.8994, 0.05}	{{1.75668, 13.8428, 0.}, {2.2194, 19.1478, 0.}}	{11.7009, 35.7095, 140.}	inf	31.	41.309	{24.1177, 55.2822}	42.2455	0.151072	newly	22.	20.46	{12., 28.}	23.4545	0.114143	Reff	1.71785	1.85124	{1.63447, 2.04634}	10.471	ar	661.	787.51	{691., 859.}	19.8351
21	R0	{1.75938, 13.5959, 2.}	{2.05771, 7.86583, 0.43}	{{1.28596, 3.26445, 0.}, {2.83858, 13.7736, 2.}}	{31.9526, 44.6531, 79.5}	inf	12.	14.9909	{4.29409, 28.5916}	63.0043	0.265468	newly	10.	24.74	{5., 54.}	165.	0.40209	Reff	1.71891	2.0016	{1.25169, 2.71323}	31.4891	ar	620.	700.57	{371., 931.}	37.5855
22	R0	{1.63456, 10.7469, 0.1}	{1.54676, 12.6527, 1.33}	{{1.27357, 8.04006, 0.}, {1.95665, 18.8228, 2.}}	{15.1726, 38.4228, 1258.}	inf	9.	12.9938	{4.09714, 20.1517}	67.5974	0.234629	newly	9.	8.94	{2., 15.}	51.7778	0.27464	Reff	1.57572	1.48362	{1.2333, 1.86224}	14.8265	ar	654.	517.57	{58., 767.}	31.2798
23	R0	{1.62702, 19.1392, 1.}	{1.54054, 7.85632, 1.09}	{{1.06738, 2.19254, 0.}, {2.31174, 16.9062, 2.}}	{26.4931, 58.9759, 113.}	inf	40.	30.2958	{6.27562, 61.19}	51.5151	0.346938	newly	13.	35.07	{15., 57.}	175.923	0.400008	Reff	1.4692	1.39743	{0.991712, 2.02955}	24.5134	ar	600.	486.77	{95., 856.}	45.115
24	R0	{1.70466, 12.1202, 2.}	{1.50797, 8.71494, 1.31}	{{1.24271, 3.26445, 0.}, {1.99038, 17.2142, 3.}}	{17.1564, 43.8303, 70.5}	inf	32.	44.5004	{12.4417, 83.973}	69.1241	0.257319	newly	23.	40.17	{18., 57.}	84.6522	0.262406	Reff	1.43191	1.24177	{1.06632, 1.55778}	16.0789	ar	613.	538.22	{301., 753.}	24.1501
25	R0	{1.61735, 11.3169, 0.1}	{1.3404, 9.1969, 0.77}	{{1.08063, 3.07157, 0.}, {1.59546, 13.1646, 2.}}	{18.0681, 33.7451, 776.}	inf	25.	9.43239	{2.25803, 15.9488}	66.0318	0.553358	newly	15.	10.65	{2., 29.}	64.6	0.441037	Reff	1.53163	1.2892	{1.04456, 1.53673}	16.7637	ar	681.	351.62	{43., 664.}	49.4156
26	R0	{1.56977, 12.3035, 2.}	{1.88174, 13.9879, 0.41}	{{1.19093, 8.35044, 0.}, {2.30108, 18.9032, 1.}}	{27.9929, 29.4757, 80.5}	inf	16.	7.98055	{2.48949, 15.2191}	55.8873	0.419144	newly	8.	5.46	{1., 11.}	59.	0.427921	Reff	1.53367	1.85248	{1.18139, 2.26488}	28.4305	ar	594.	652.65	{30., 879.}	40.0993
27	R0	{1.46317, 5.8298, 0.1}	{2.47512, 13.4342, 0.5}	{{1.96658, 9.82151, 0.}, {2.93371, 18.0404, 1.}}	{69.1613, 130.44, 502.}	inf	8.	53.6255	{29.7344, 78.2754}	570.319	0.796143	newly	8.	38.84	{24., 51.}	385.5	0.666897	Reff	1.39294	2.23887	{1.84673, 2.62168}	60.7302	ar	586.	875.39	{788., 936.}	49.384
28	R0	{1.70002, 14.9806, 1.}	{1.72607, 12.1672, 0.97}	{{1.07874, 3.98513, 0.}, {2.32891, 18.765, 2.}}	{25.3822, 31.6408, 67.}	inf	4.	5.27879	{1.71142, 10.9268}	67.1835	0.243258	newly	0.1	5.63	{0., 13.}	5550.	1.44191	Reff	1.68812	1.71084	{1.07325, 2.3028}	25.1481	ar	652.	492.62	{7., 867.}	48.7393
29	R0	{1.52729, 16.1014, 3.}	{1.90235, 15.6775, 0.59}	{{1.43451, 12.7642, 0.}, {2.22832, 19.5406, 1.}}	{26.3743, 15.6005, 80.3333}	inf	40.	33.3126	{17.8557, 47.7538}	25.7458	0.144487	newly	22.	17.33	{11., 25.}	26.5	0.144263	Reff	1.40358	1.75888	{1.35714, 2.01908}	26.6452	ar	592.	741.37	{559., 856.}	30.4949
30	R0	{1.72795, 13.5635, 1.}	{1.23806, 5.66173, 1.1}	{{1.06738, 2.19254, 0.}, {1.44466, 12.6754, 4.}}	{28.6098, 59.733, 128.}	inf	42.	17.0883	{4.56089, 34.3164}	60.8843	0.520545	newly	22.	27.16	{7., 60.}	68.2727	0.28773	Reff	1.5396	1.13254	{0.979256, 1.31492}	26.6253	ar	668.	323.7	{91., 551.}	52.1647
31	R0	{1.51591, 18.1544, 3.}	{1.77892, 12.643, 0.85}	{{1.38815, 6.71734, 0.}, {2.20181, 18.6082, 2.}}	{22.0453, 31.0813, 71.6667}	inf	41.	27.2157	{10.6719, 40.6283}	37.9646	0.254035	newly	22.	19.14	{9., 30.}	35.7273	0.205557	Reff	1.4007	1.66544	{1.31634, 2.06026}	22.5418	ar	541.	667.28	{430., 856.}	36.8318
32	R0	{1.4282, 10.3534, 0.1}	{2.14247, 15.5356, 0.47}	{{1.63746, 10.1332, 0.}, {2.88523, 19.4475, 1.}}	{50.1142, 51.5707, 496.}	inf	4.	17.9176	{6.59267, 31.4051}	348.1	0.589104	newly	3.	10.1	{4., 18.}	242.	0.474693	Reff	1.40535	2.07557	{1.60702, 2.75206}	47.8127	ar	521.	799.59	{644., 930.}	55.8215
33	R0	{1.56693, 11.3972, 0.1}	{2.06486, 11.2928, 0.86}	{{1.28596, 4.06661, 0.}, {2.86095, 18.33, 2.}}	{39.6097, 35.9054, 848.}	inf	11.	9.84047	{2.68993, 20.6204}	50.463	0.270495	newly	6.	11.4	{2., 30.}	128.	0.372633	Reff	1.54029	2.03042	{1.26608, 2.81445}	39.4454	ar	695.	685.7	{179., 930.}	33.0532
34	R0	{1.50675, 19.8823, 1.}	{1.3952, 7.04674, 1.03}	{{1.22544, 3.07157, 0.}, {1.70173, 15.1079, 3.}}	{15.4668, 64.5577, 117.}	inf	62.	34.5344	{8.6486, 66.6928}	49.5426	0.405179	newly	30.	39.17	{14., 60.}	49.5667	0.193002	Reff	1.22047	1.16387	{1.02658, 1.37525}	12.3229	ar	587.	450.95	{203., 706.}	33.9608
35	R0	{1.72889, 7.76068, 0.1}	{2.32557, 9.18003, 0.4}	{{1.47488, 4.00571, 0.}, {2.9194, 13.3345, 1.}}	{39.4479, 38.5965, 440.}	inf	6.	13.8548	{3.9432, 25.2983}	143.683	0.354723	newly	7.	19.53	{5., 36.}	191.286	0.412267	Reff	1.70469	2.27305	{1.44836, 2.85944}	38.3609	ar	650.	802.2	{548., 940.}	34.9969
36	R0	{1.62644, 4.12303, 0.1}	{2.13493, 10.1974, 0.69}	{{1.39972, 4.66877, 0.}, {2.8186, 16.0359, 2.}}	{38.2772, 151.563, 692.}	inf	5.	5.51985	{1.70494, 9.4293}	54.5591	0.240599	newly	11.	10.59	{1., 20.}	94.6364	0.512742	Reff	1.61343	2.11613	{1.38709, 2.78914}	38.1378	ar	652.	709.2	{21., 928.}	37.8374
37	R0	{1.70878, 15.8072, 1.}	{1.52906, 12.789, 1.61}	{{1.34829, 8.35044, 0.}, {1.95665, 16.748, 3.}}	{16.3758, 22.8826, 107.}	inf	53.	36.4102	{18.1463, 65.2006}	41.1855	0.261505	newly	18.	22.04	{12., 35.}	42.7778	0.168893	Reff	1.48835	1.35371	{1.21034, 1.67681}	14.3186	ar	674.	547.77	{369., 772.}	25.0668
38	R0	{1.58967, 6.80568, 0.1}	{2.40072, 11.0994, 0.33}	{{1.737, 5.83416, 0.}, {2.90165, 15.3681, 1.}}	{52.586, 69.2634, 366.}	inf	6.	17.4204	{6.69194, 27.8646}	197.274	0.430441	newly	10.	18.03	{6., 34.}	101.9	0.29828	Reff	1.55947	2.32583	{1.67999, 2.80572}	50.7539	ar	610.	845.81	{667., 928.}	41.2213
39	R0	{1.344, 8.19498, 0.1}	{1.7567, 14.7447, 1.48}	{{1.38427, 10.1332, 0.}, {2.12978, 18.7084, 3.}}	{32.2041, 84.0216, 1418.}	inf	10.	24.9371	{11.1136, 38.3357}	155.345	0.382889	newly	8.	13.84	{5., 24.}	86.25	0.258246	Reff	1.2889	1.65614	{1.31792, 1.9899}	30.019	ar	551.	676.99	{453., 830.}	31.8348
40	R0	{1.61509, 7.90825, 0.1}	{1.9555, 13.6169, 0.45}	{{1.18158, 8.71343, 0.}, {2.80387, 18.7523, 1.}}	{33.1749, 77.0607, 482.}	inf	9.	3.46266	{1.26825, 6.90816}	62.3179	0.496848	newly	10.	3.28	{0., 6.}	77.8	0.857552	Reff	1.59894	1.94614	{1.17809, 2.7796}	33.4587	ar	581.	575.64	{3., 922.}	55.4492
41	R0	{1.60017, 5.90605, 0.1}	{2.33489, 13.9989, 0.28}	{{1.74592, 9.08517, 0.}, {2.88523, 18.4042, 1.}}	{46.7731, 137.403, 330.}	inf	8.	16.714	{7.36113, 26.3497}	116.524	0.317662	newly	8.	12.33	{3., 22.}	74.875	0.245485	Reff	1.56817	2.26649	{1.70808, 2.77015}	45.3794	ar	661.	839.65	{715., 932.}	29.2088
42	R0	{1.64436, 15.1337, 1.}	{2.03715, 11.5452, 1.4}	{{1.06738, 2.5149, 0.}, {2.76849, 18.9813, 2.}}	{37.8891, 36.0656, 98.}	inf	21.	32.3449	{9.12518, 58.9693}	83.3452	0.29209	newly	9.	28.71	{13., 51.}	220.333	0.455461	Reff	1.5605	1.90108	{1.03067, 2.59586}	35.4511	ar	640.	700.36	{267., 927.}	36.5219
43	R0	{1.48917, 11.917, 0.1}	{1.63145, 15.07, 0.39}	{{1.4874, 12.483, 0.}, {1.76949, 19.2972, 2.}}	{10.6879, 28.8758, 442.}	inf	31.	20.8512	{11.6588, 28.7121}	34.4159	0.205903	newly	19.	11.07	{6., 18.}	43.1053	0.284337	Reff	1.38791	1.53683	{1.40667, 1.67273}	11.6112	ar	551.	638.66	{496., 752.}	21.9964
44	R0	{1.65577, 7.89317, 1.}	{2.24354, 12.8133, 0.67}	{{1.59621, 6.0871, 0.}, {2.93488, 18.8445, 2.}}	{39.8698, 69.487, 69.}	inf	6.	13.2286	{3.97062, 23.6382}	134.471	0.343616	newly	9.	11.57	{3., 23.}	69.6667	0.286648	Reff	1.63093	2.19187	{1.5675, 2.84784}	38.7596	ar	576.	802.13	{619., 932.}	47.3524
45	R0	{1.6909, 12.3275, 0.1}	{1.86064, 11.3209, 0.84}	{{1.3618, 4.00571, 0.}, {2.315, 19.0355, 2.}}	{21.2467, 36.3346, 826.}	inf	23.	35.42	{12.641, 58.5107}	73.2858	0.244191	newly	14.	28.66	{12., 44.}	110.571	0.295031	Reff	1.5793	1.7088	{1.2858, 2.11359}	19.2314	ar	675.	698.83	{446., 878.}	22.0578
46	R0	{1.62351, 16.0698, 2.}	{1.60325, 11.6092, 0.63}	{{1.13227, 5.71232, 0.}, {1.95665, 17.7016, 2.}}	{18.5275, 32.2387, 70.5}	inf	17.	16.8728	{4.62425, 32.2114}	48.3149	0.253813	newly	10.	11.84	{4., 19.}	45.	0.190571	Reff	1.55532	1.53359	{1.09331, 1.86535}	17.8701	ar	591.	549.68	{40., 792.}	36.1083
47	R0	{1.52372, 4.38599, 0.1}	{2.07921, 9.76958, 0.76}	{{1.38815, 4.02511, 0.}, {2.86095, 16.748, 2.}}	{42.095, 127.014, 756.}	inf	3.	5.65591	{1.56883, 10.5957}	107.288	0.293466	newly	3.	11.54	{1., 20.}	316.	0.530744	Reff	1.51458	2.06035	{1.37746, 2.82004}	41.7012	ar	618.	686.37	{18., 931.}	42.4547
48	R0	{1.44306, 11.788, 1.}	{2.26672, 14.8309, 0.19}	{{1.81647, 10.5184, 0.}, {2.93978, 19.9817, 1.}}	{57.1344, 32.8304, 81.}	inf	30.	58.6264	{27.675, 91.7876}	103.255	0.287398	newly	21.	33.87	{16., 50.}	70.3333	0.226037	Reff	1.32617	2.00944	{1.65179, 2.49347}	51.5289	ar	534.	831.99	{708., 930.}	56.0768
49	R0	{1.63624, 11.3893, 1.}	{2.28383, 13.031, 0.87}	{{1.58886, 6.00596, 0.}, {2.86501, 18.1089, 2.}}	{42.7307, 32.1578, 61.}	inf	10.	15.337	{6.43385, 27.2752}	69.7792	0.224027	newly	9.	12.98	{3., 23.}	79.1111	0.278533	Reff	1.60188	2.2223	{1.53928, 2.78698}	41.9135	ar	643.	818.96	{674., 929.}	34.0684
50	R0	{1.46113, 11.1322, 1.}	{1.86307, 13.8047, 0.88}	{{1.39882, 5.43578, 0.}, {2.24242, 18.8819, 2.}}	{30.0411, 43.6938, 68.}	inf	12.	13.1026	{4.83453, 21.9984}	47.7403	0.214809	newly	6.	8.92	{2., 15.}	79.6667	0.268575	Reff	1.4246	1.81303	{1.36288, 2.19656}	29.6448	ar	501.	701.31	{474., 865.}	49.5429
